# Regulatory Issues in Electronic Health Records for Adolescent HIV Research: Strategies and Lessons Learned

**DOI:** 10.2196/46420

**Published:** 2024-05-02

**Authors:** Sara Shaw Green, Sung-Jae Lee, Samantha Chahin, Meardith Pooler-Burgess, Monique Green-Jones, Sitaji Gurung, Angulique Y Outlaw, Sylvie Naar

**Affiliations:** 1 Center for Translational Behavioral Science Department of Behavioral Sciences and Social Medicine Florida State University Tallahassee, FL United States; 2 Division of Population Behavioral Health Department of Psychiatry and Biobehavioral Sciences University of California, Los Angeles Los Angeles, CA United States; 3 College of Liberal Arts and Sciences Wayne State University Detroit, MI United States; 4 Department of Health Sciences New York City College of Technology New York, NY United States; 5 Division of Behavioral Health, Department of Family Medicine and Public Health Sciences College of Medicine Wayne State University Detroit, MI United States

**Keywords:** electronic health record, HIV, pragmatic trial, regulatory, EHR, pre-exposure prophylaxis, retention, attrition, dropout, legal, regulation, adherence, ethic, review board, implementation, data use, privacy

## Abstract

**Background:**

Electronic health records (EHRs) are a cost-effective approach to provide the necessary foundations for clinical trial research. The ability to use EHRs in real-world clinical settings allows for pragmatic approaches to intervention studies with the emerging adult HIV population within these settings; however, the regulatory components related to the use of EHR data in multisite clinical trials poses unique challenges that researchers may find themselves unprepared to address, which may result in delays in study implementation and adversely impact study timelines, and risk noncompliance with established guidance.

**Objective:**

As part of the larger Adolescent Trials Network (ATN) for HIV/AIDS Interventions Protocol 162b (ATN 162b) study that evaluated clinical-level outcomes of an intervention including HIV treatment and pre-exposure prophylaxis services to improve retention within the emerging adult HIV population, the objective of this study is to highlight the regulatory process and challenges in the implementation of a multisite pragmatic trial using EHRs to assist future researchers conducting similar studies in navigating the often time-consuming regulatory process and ensure compliance with adherence to study timelines and compliance with institutional and sponsor guidelines.

**Methods:**

Eight sites were engaged in research activities, with 4 sites selected from participant recruitment venues as part of the ATN, who participated in the intervention and data extraction activities, and an additional 4 sites were engaged in data management and analysis. The ATN 162b protocol team worked with site personnel to establish the necessary regulatory infrastructure to collect EHR data to evaluate retention in care and viral suppression, as well as para-data on the intervention component to assess the feasibility and acceptability of the mobile health intervention. Methods to develop this infrastructure included site-specific training activities and the development of both institutional reliance and data use agreements.

**Results:**

Due to variations in site-specific activities, and the associated regulatory implications, the study team used a phased approach with the data extraction sites as phase 1 and intervention sites as phase 2. This phased approach was intended to address the unique regulatory needs of all participating sites to ensure that all sites were properly onboarded and all regulatory components were in place. Across all sites, the regulatory process spanned 6 months for the 4 data extraction and intervention sites, and up to 10 months for the data management and analysis sites.

**Conclusions:**

The process for engaging in multisite clinical trial studies using EHR data is a multistep, collaborative effort that requires proper advanced planning from the proposal stage to adequately implement the necessary training and infrastructure. Planning, training, and understanding the various regulatory aspects, including the necessity of data use agreements, reliance agreements, external institutional review board review, and engagement with clinical sites, are foremost considerations to ensure successful implementation and adherence to pragmatic trial timelines and outcomes.

## Introduction

Electronic health records (EHRs) can serve as a useful tool to inform various outcomes related to HIV prevention and care among minoritized youth. For example, Dark et al [1] assessed the feasibility of using EHRs to examine and monitor the HIV care continuum and costs associated with implementation science studies for youth living with HIV. In addition, Butame et al [2] highlighted barriers and facilitators to the collection of aggregate HIV-related EHR data. In the field of HIV prevention, Dunn et al [3] used EHRs to capture primary HIV prevention outcomes. In the field of sexually transmitted infections, Jackman et al [4] leveraged EHRs to reduce disparities in the rates of sexually transmitted infections among university students.

EHRs can provide a foundation for innovative, large-scale simple trials at a lower cost than traditional randomized controlled trials and ultimately achieve a learning health care system [5]. While an explanatory (or efficacy) trial seeks to determine if an intervention is effective under ideal conditions to maximize its favorable effects, a pragmatic trial aims to demonstrate that an intervention can be effective in real-world settings. Pragmatic trials often enroll patients most in need, test treatments within settings and with staff who are typical to most clinical situations, and address issues important to clinicians, policy makers, and patients [6]. Furthermore, embedding pragmatic trials within clinical care settings can facilitate the investigation of treatment effects in a more cost-efficient manner than trials conducted without the clinical infrastructure provided by such a setting [7]. These types of trials are perfectly suited for clinic-level interventions focusing on retention in pre-exposure prophylaxis (PrEP) and HIV treatment services.

The Adolescent Trials Network (ATN) for HIV/AIDS Interventions Protocol 162b (ATN 162b) sought to pilot a pragmatic approach to evaluate mobile health (mHealth) retention in a care intervention for PrEP and antiretroviral therapy (ART) using EHRs to determine improvements in on-site appointments arranged across patients without having to obtain consent and collect data from individual patients. Additional EHR variables were collected as a pilot for continua monitoring in future trials as evidence-based guidelines include recommendations for systematic monitoring of HIV care continuum variables such as linkage and retention in care and ART adherence [8,9] and monitoring of the PrEP continuum [10]. These studies also served as a pilot for routine EHR downloads to monitor HIV prevention and treatment continua at ATN sites.

Despite the simplification of the consent process within pragmatic trials [11], there are significant regulatory requirements and associated challenges that may emerge in such trials, including the establishment of reliance and data use agreements [12] ensuring deidentification of individual intervention and outcome data [13], responsiveness to changing regulatory requirements as researchers and institutions respond to everchanging technology and related bioethical concerns [14], and compliance with sponsor data sharing practices [15]. Experts have identified multiple key ethical and regulatory challenges unique to pragmatic trials, including the necessity for harmonization among collaborating institutional review boards (IRBs) [16], and the need to streamline the regulatory oversight process [14,16]. Based on these identified challenges, efforts have been made in recent years to enhance the collaborative nature of multisite clinical trial research such as the policy issued regarding the requirement of a single IRB for National Institutes of Health–funded projects [17]; however, these policy changes are still in their infancy and contain inconsistencies, resulting in confusion regarding their implementation and delays that may impact study timelines. The objective of this study is to describe the unique regulatory processes, challenges, strategies, and lessons learned in the implementation of a multisite pragmatic trial with EHR outcomes in the ATN 162b to contribute useful recommendations for future researchers conducting such trials.

## Methods

### Overview

The ATN 162b used a quasi-experimental longitudinal design to collect annual EHR via data downloads, supplement with auxiliary data, and curate and harmonize the data using a common data model to support the measurement of retention in care and viral suppression for pilot-testing an mHealth intervention. Para-data, such as usage metrics from components of the intervention app, were also collected to assess the feasibility and acceptability of the intervention [18]. A PrEP site and an HIV treatment site participated in the interventions and another PrEP site and HIV treatment site served as controls. All youth aged 18-24 years enrolled at the 2 sites were eligible for participation.

The process for EHR data extraction required significant attention to the types of data to be extracted and transferred, establishment of the necessary regulatory agreements, a detailed protocol to be developed, and participating sites to be trained. A limited data set (deidentified except for dates of service) was extracted from 4 ATN participant recruitment venues longitudinally, representing 2 full years (2018-2019). Demographics, behavioral measures, clinical data, prevention services data, and linkage- and retention-in-care data were extracted from EHRs and electronic appointment systems supplemented with remote data entry with all identifiers removed except for dates of service. Data were managed and analyzed in a secure cloud database. Clinic-level data were used as pragmatic outcomes for a pilot study of mHealth apps prevention and treatment, and not to analyze how the intervention impacted health or behavioral outcomes in human participants; hence, the study did not need to be registered as a clinical trial [19].

Sites were trained in a standard protocol to electronically download data, which reduces the risk of data entry error, particularly for complex variables (eg, *ICD-10* [*International Statistical Classification of Diseases, Tenth Revision*] codes). Exceptions for manually extracted variables (eg, laboratory findings from other sites or other paper records) were made so that sites could enter data directly into a supplemental database using an appropriate electronic data collection system (eg, Conserved Domain Architecture Retrieval Tool) [20]. Participant recruitment venues were provided training and an extraction procedural package that included a study summary, data explanation form, protocol adherence checklist, launch email, database user account information, and the data files request link for each site.

Data were directly transferred by a trained site coordinator annually (2018 and 2019) to the coordinating center in accordance with established file transfer protocols and stored using HIPAA (Health Insurance Portability and Accountability Act)-compliant cloud computing services. Once securely stored, data were curated and harmonized by the study’s data management team. To prevent protocol violations and the inadvertent upload of patient data that were not covered as part of the protocol, each site submitted a protocol adherence checklist to the protocol team, who approved it before any data uploads occurred. This mandatory checklist served to ensure multiple reviews of the final data set before transmitting data.

An existing evidenced-based youth HIV adherence intervention, Motivational Enhancement System for Adherence (MESA) [21], was refined for adherence to PrEP appointments and HIV treatment appointments. MESA is built on a novel, low-cost intervention platform, Computerized Intervention Authoring Software, which allows investigator-initiated intervention development without programming, thus dramatically reducing the time and cost of reducing ideas to a usable, editable, and shareable application. MESA uses a digital counselor, an animated avatar of the youth’s choosing, to deliver motivational interviewing and goal setting for a specific target behavior. Intervention content was refined on the basis of existing ATN studies and the ATN’s youth advisory groups. The interventions were delivered via a tablet during the clinic visit, at baseline, and at the time of the next scheduled usual care appointment. To ensure anonymity for this partially deidentified data-driven pragmatic feasibility trial, youth will use a “burner” app that provides a second phone number to receive text messages. The site coordinator approached all eligible youth, completed onboarding, assisted participants in downloading the “burner” app with notifications turned on, and assisted them with accessing the web-based intervention on the provided tablet. Youth also received disposable earbuds and then independently completed a single 15-20–minute MESA session while at the clinic. At the end of the session, the site coordinator entered the youths’ next clinic appointment into the intervention application and provided them US $10 in the form of cash or a gift card. Participants then received monthly automated motivational reinforcement and visit text message reminders for their next appointment. At the next appointment (usually quarterly at adolescent sites), the site coordinators guided youth to complete a brief 5-minute follow-up MESA session and receive a second, final US $10 payment.

### Ethical Considerations

This study was reviewed and approved by the Florida State University (FSU) IRB (STUDY00000619), the single IRB of record (sIRB). Informed consent was obtained from those individuals who participated in the intervention activities, as informed consent was not required for acquiring the limited data set in line with the HIPAA privacy rule for a covered entity [22]. Acquisition of the limited data set was secured by executing data use agreements, and data were shared as password-protected files on a secure cloud server. As stated above, those participating in the intervention activities were eligible to receive up to US $20 either as cash or gift cards.

### Measures

#### Prevention Variables

Descriptive variables obtained from EHRs with regard to HIV prevention among adolescents in the ATN 162b were the visit date; current age; date of birth; gender; sexual orientation; race; ethnicity; 3 levels of PrEP; indicators of PrEP eligibility, acceptance, and status; behavioral risk factors; smoking status; substance abuse; behavioral health status; mental health; sexual risk; results of tests for sexually transmitted infections; other medical statuses; height and weight; BMI; and results of pregnancy testing.

Descriptive variables obtained from EHRs with regard to HIV care services for adolescents in the ATN 162b include HIV testing; risk assessment; HIV care referral; PrEP referral; a Centers for Disease Control and Prevention–mandated checklist for PrEP providers (such as providing basic education about PrEP; obtaining past medical history including kidney and liver [ie, hepatitis] disease, bone disease, and fractures, as well as assessment of pregnancy desires for women; assessing for possible symptoms of acute HIV infection; performing laboratory tests including HIV tests to rule out acute HIV infection, tests for sexually transmitted diseases, tests for serum creatinine to determine creatinine clearance, hepatitis B surface antigen and antibody, and hepatitis C virus antibody, body weight for determining creatinine clearance, and pregnancy tests; providing prescriptions for Truvada [30 tablets]; providing PrEP education or counseling; assessing substance abuse and mental health needs and make referrals as appropriate; reviewing the importance of regular clinic follow-up and determining the best method of communication for reminders [eg, call, email, or SMS text message]; scheduling follow-up visits for 1 month and providing an appointment card; and starting hepatitis B vaccine series and administering meningococcal and human papillomavirus vaccines as appropriate); follow-up visits (30 days after the initial visit and quarterly thereafter; including PrEP adherence monitoring during provider visits [with dried blood spots at provider visits]); behavioral health referral; behavioral health visits; outreach, advocacy, and case management visits; testing for sexually transmitted infections; and pregnancy testing.

#### Care Variables

Descriptive variables obtained from EHRs with regard to HIV care among adolescents in the ATN 162b include the visit date; current age; date of death; gender; sexual orientation; race; ethnicity; diagnostic characteristics; age at HIV diagnosis; date of HIV diagnosis; mode of transmission; 3 levels of ART; indicators of indicators of ART eligibility, acceptance, and status; HIV treatment; date of entry into care; names of antiretroviral medications; viral load results; CD4^+^ lymphocyte count; names of other non-HIV medications; behavioral risk factors; smoking status; substance abuse; behavioral health status; mental health; sexual risk; results of tests for sexually transmitted infections; other medical statuses; *ICD-10* diagnosis codes or other indicators of medical mental health or substance abuse; height and weight; BMI; blood pressure; cholesterol panel; blood glucose; and pregnancy test results.

Variables obtained from EHRs with regard to HIV care services for adolescents in the ATN 162b include CPT-4 (Current Procedural Terminology), HCPCS (Healthcare Common procedure Coding System), or other indicators of medical and behavioral provisions; risk assessment; ART referral; medical visits attended (for retention in care); medical visits missed (for visit consistency); behavioral health referrals (eg, mental health and substance abuse treatment); behavioral health visits; outreach, advocacy, and case management visits; testing for sexually transmitted infections (*Neisseria gonorrhoeae* and *Chlamydia trachomatis* in urine, the rectum, and pharynx, and rapid plasma reagin); and pregnancy testing.

## Results

Our results focus on the unique regulatory processes implemented by the ATN 162b team, which are related to the (1) development and coordination of the necessary regulatory infrastructure across multiple academic and clinic sites, (2) preparing for and initiating site-specific training of study personnel, (3) ongoing monitoring of regulatory components, and (4) ensuring compliance with institutional and sponsor requirements.

Regulatory aspects of developing a multisite study focused on the extraction and analysis of limited data sets require significant attention to regulatory compliance. Regarding the split approach of protocol development, consent for the limited data set was not required from the study participants, as the protocol included limited data sets only and was considered to be of minimal risk; however, HIPAA authorization was required for extracting these data and encompassed all 4 participating sites, in line with the US Department of Health and Human Services guidelines related to the use of Protected Health Information for the purpose of research [9]. For sites participating in the study intervention, informed consent was obtained through a standard process with site-specific documents. Based on these distinctions, to accomplish the regulatory components, study personnel at FSU, as the sIRB, were tasked with the development of reliance agreements and data use agreements that differed depending on the site’s data versus intervention activities. The timing of the development and execution of these agreements posed challenges to the initiation of the study protocol, as clinical sites had varying requirements related to their internal regulatory processes and authorized signatory needs. The process for regulatory development began in the fall of 2019, with the submission of the study to the overseeing sIRB at FSU. As indicated in Multimedia Appendices 1 and 2, three of the 4 sites that participated in the pragmatic trial and in collecting EHRs and those that only collected EHRs were initiated (light gray) in June 2020, and there was considerable variance in the full execution (dark gray). Data use agreements were initiated and executed with data analysis sites as data were not intended to be transferred from the data management site until the completion of the study phases.

Once the protocol was submitted and approved as a multisite study, the project team worked to finalize the participating sites’ locations and worked with the sIRB to determine the appropriate regulatory components for the study (eg, reliance and data use agreements). Ultimately, the sIRB determined that all sites were considered to have been engaged in human subjects research, regardless of whether they were intervention or nonintervention and data extraction sites. Hence, the study team chose to separate the protocol into 2 parts: the first part focused on intervention-only sites, and the second part addressing data extraction separately. This split was intended to allow for the development of informational and instructional materials tailored to each type of clinical site to ensure adequate training of site personnel and to avoid any overlap or confusion among the pragmatic trial intervention and nonintervention sites. Furthermore, this distinction allowed the study team to ensure that proper monitoring of protocol-specific activities could be conducted, and necessary modifications or reports of information to the IRB were specific to the type of activities, as distinguished between the intervention and nonintervention sites. In addition to intervention and nonintervention sites, reliance and data use agreements were executed with external collaborators on the data management team to ensure compliant access to the limited data set. Ongoing monitoring of all relevant protocols and site activities was the responsibility of the sIRB site (FSU), including the submission of modifications, as well as close collaboration with the ATN Coordinating Center, to ensure that data storage and management aligned with the provisions of the data use agreements and complied with the sponsors’ requirements related to data sharing.

## Discussion

### Principal Findings

This paper describes the regulatory processes, challenges, and strategies to navigate them, and lessons learned in the ATN 162b multisite embedded pragmatic trial within HIV testing and prevention clinical sites to evaluate clinical-level outcomes related to HIV treatment or PrEP services.

The regulatory processes of the project described in this paper, such as ceding of IRB oversight to the sIRB, development and coordination of the regulatory infrastructure, training of personnel, and execution of necessary regulatory agreements, were not without their challenges. For example, different sites had different requirements when executing reliance agreements, often requiring a full submission to their own internal IRB for review before reliance agreements could be executed. Specifically, although the sIRB determined that informed consent is not required in accordance with the US Department of Health and Human Research policy [15], the intervention sites determined that informed consent was needed on the basis of their own internal review, which added additional regulatory components (site specific consent forms) and subsequent regulatory processes in the establishment of the necessary agreements. Based on these external processes, the study team was faced with significant delays at the start of intervention and data extraction training and had to address site-specific requirements related to HIPAA authorization, consent, etc. Additionally, these inconsistencies with site-specific determinations and subsequent delays impacted the timeliness of personnel training across the participating sites, further delaying the implementation of the intervention’s activities.

### Strategies and Lessons Learned

The execution of this pilot yielded several regulatory lessons that allowed for the successful and compliant implementation of a pilot pragmatic trial and paved the way for a successful full-scale trial. Strategies used by the study team to navigate regulatory challenges include (1) adopting a phased approach to protocol development and review of regulatory processes to allow for more effective multisite collaboration and ensure compliance with the study’s timeline; (2) coordinating appropriate training of multisite personnel specific to their role on the team, which varied greatly based on site-specific activities; and (3) ensuring that all regulatory aspects were coordinated and monitored by a specialized team member at the sIRB, who served as a primary point of contact across all sites and provided consistent, clear, and timely information related to all regulatory components and requirements.

The regulatory lessons learned from this multisite study indicate a need for investigators to prepare as early as possible owing to the collaborative nature of multisite human subjects research—for example, as early as the grant preparation phase—and gain a general understanding of all participating sites’ unique regulatory requirements to adequately prepare for entering into the necessary agreements to begin the research. Having this understanding as part of the grant proposal phase will undoubtedly assist in the development and execution of these agreements upon receiving a notice of award, and reduce the likelihood of overall study timeline delays caused by delayed establishment of the regulatory infrastructure. Additionally, we recommend having skilled individuals appointed to the project who are tasked specifically with the regulatory management of multisite trials. While ultimately, it is the responsibility of the study’s principal investigator to oversee all regulatory components, having dedicated personnel who are knowledgeable in the nuances of regulatory aspects of clinical trial research and experienced in developing multisite agreements and establishing multisite infrastructure in line with these agreements would further prevent any delays in the ability to begin study activities.

### Contribution to the Existing Literature

Much of the existing literature that describes similar studies more often focuses on aspects related to the study design and methodology of embedded pragmatic trials and addresses ethical considerations through blanket statements that include IRB review, compensation, informed consent, etc [7,23,24]. Further, literature focused on providing recommendations to researchers or regulatory bodies (or both) on the challenges and recommendations related to multisite research [12,25] and pragmatic trials [14,16] has been effective in implementing new approaches in regulatory processes, such as central IRB review and oversight and providing standardized methods for promoting multisite collaborations; such literature is more often generalizable, rather than providing practical examples within the context of a specific pragmatic trial in detail. Thus, the literature on regulatory elements required for conducting multisite pragmatic research and timelines for ensuring regulatory components is scarce, particularly after the implementation of the sIRB policy enacted in 2016 [17]. When considering existing literature that focuses on the ethical considerations of pragmatic trial research as it relates to EHR data, much of this work tends to focus on the larger ethical implications of data sharing and informed consent rather than the regulatory aspects required of researchers undertaking this work [24,26]. Thus, this paper uniquely describes the procedures and the lessons learned from the perspective of a specific project that may serve as a framework for future studies.

### Limitations

The regulatory outcomes of the study are not without their limitations. First, the regulatory elements specific to this project are undoubtedly not specific to pragmatic trials. Further, as this study was part of the ATN, these regulatory findings may not be generalizable to other multisite trials that do not operate within a larger federally funded network structure, with their own data sharing and management expectations and policies. Additionally, the protocols were impacted by the onset of the COVID-19 pandemic. These delays resulted in modifications of EHR data downloads, limiting the data to 2018 and 2019, as opposed to the original plan to download 2018-2021 EHR data. In addition, the pragmatic trial’s timeline was significantly impacted by the COVID-19 pandemic, particularly for those intervention sites where direct participant engagement occurred, wherein the sIRB required modifications to the study protocol that included COVID-19 mitigation practices as they relate to participant safety.

### Conclusions

The necessary regulatory components of conducting multisite embedded pragmatic trials require considerable planning on behalf of researchers from the grant proposal stage through study completion. Researchers must consider realistic timelines, necessary regulatory infrastructure associated with the use of EHR data, understanding the requirements for complying with guidance from federal sponsors (eg, the National Institutes of Health) related to data sharing and management, and adequate preparation and training of project personnel. Future pragmatic trials should provide further details for regulatory processes, challenges, and timelines to expand on the literature in this domain.

## Appendix

Multimedia Appendix 1 Timeline for multisite reliance agreements across ATN 162b (Adolescent Trials Network for HIV/AIDS Interventions Protocol 162b) prevention, treatment, and data collection sites.

Multimedia Appendix 2 Timeline for multisite data use agreements for electronic health records for adolescent HIV research.

## Abbreviations

ART: antiretroviral therapy
ATN: Adolescent Trials Network
ATN 162b: Adolescent Trials Network for HIV/AIDS Interventions Protocol 162b
CPT-4: Current Procedural Terminology
EHR: electronic health record
FSU: Florida State University
HCPCS: Healthcare Common procedure Coding System
HIPAA: Health Insurance Portability and Accountability Act
*ICD-10*: *International Statistical Classification of Diseases, Tenth Revision*
IRB: institutional review board
MESA: Motivational Enhancement System for Adherence
mHealth: mobile health
PrEP: pre-exposure prophylaxis
sIRB: single institutional review board of record

## Data Availability

Data associated with the ATN 162b (Adolescent Trials Network for HIV/AIDS Interventions Protocol 162b) are held by the primary study site (Florida State University) and will be published in the main study manuscript (under preparation). Researchers may contact the study’s principal investigator to make specific data requests.
