# Supplementary material for: Regulatory Issues in Electronic Health Records for Adolescent HIV Research: Strategies and Lessons Learned
Source: JMIR Form Res. 2024 May 2;8:e46420. doi: 10.2196/46420 (PMC11099806; doi:10.2196/46420)
Supplement: Multimedia Appendix 1 [file formative_v8i1e46420_app1.docx]

Timeline for multisite reliance agreements across ATN 162b (Adolescent Trials Network for HIV/AIDS Interventions Protocol 162b) prevention, treatment, and data collection sites.

| **Reliance Agreements Timeline** | | | | | | | | | | | | | |
| --- | --- | --- | --- | --- | --- | --- | --- | --- | --- | --- | --- | --- | --- |
| **Year** | **2020** | | | | | | | | **2021** | | | | |
| **Quarter** | **Q2** | | **Q3** | | | **Q4** | | | **Q1** | | **Q2** | | |
| PT + EHR Prevention Site 1 |  |  |  | | |  |  |  |  | |  | | |
| PT + EHR Treatment Site 1 |  | |  | | |  |  |  |  | |  | | |
| EHR Prevention Site 2 |  | |  | | |  | | |  |  |  |  |  |
| EHR Treatment Site 2 |  |  |  |  |  |  | | |  | |  | | |
| Data Management Site |  |  |  | | |  | | |  |  |  | | |
| Data Analysis Site 1 |  |  |  |  |  |  | | |  | |  | | |
| Data Analysis Site 2 |  | |  | | |  | | |  |  |  |  |  |
| Data Analysis Site 3 |  | |  | | |  | | |  |  |  |  | |
| Intervention Management Site |  | |  | | |  | | |  |  |  |  | |
